# Effect of Fiber Mass Fraction on Microstructure and Properties of 2D CF-GO/EP Composite Prepared by VIHPS

**DOI:** 10.3390/nano12071184

**Published:** 2022-04-01

**Authors:** Yuqin Ma, Yi Chen, Fei Li, Yiren Xu, Wei Xu, Yatao Zhao, Haiyin Guo, Yatao Li, Zedu Yang, Yi Xu

**Affiliations:** 1 State Key Laboratory of Public Big Data, Guizhou University, Guiyang 550025, China; 2 School of Mechano-Electronic Engineering, Xidian University, Xi’an 710071, China; xdchenyi@163.com (Y.C.); lifeili1123@163.com (F.L.); weix@xidian.edu.cn (W.X.); xidianyatao@163.com (Y.Z.); haiyin_guo@163.com (H.G.); leeyetee@gmail.com (Y.L.); y487800984@163.com (Z.Y.); yixu6399267@163.com (Y.X.); 3 School of Mechanical Engineering, Tsinghua University, Beijing 100084, China

**Keywords:** fiber mass fraction, graphene oxide, carbon fiber composites, flexural strength, interlaminar fracture toughness

## Abstract

Graphene is often used to improve interlaminar fracture toughness of carbon fiber/epoxy resin (CF/EP) composites. It is still a challenge to improve the toughness while maintaining the in-layer properties. In this study, 2D graphene oxide carbon fiber reinforced epoxy resin matrix (2D CF-GO/EP) composites were prepared by a vacuum infiltration hot-press forming experimental system (VIHPS), and three-point flexural and end notch flexural (ENF) tests were carried out. With the increase of the fiber mass fraction in the composites, the mode II interlaminar fracture toughness (*G*_II__C_) layers decrease gradually, and the bond property between the fiber and matrix interface layer becomes worse, because the accumulation of dense fiber bundles reduces the matrix penetration ability of cracks. However, the flexural properties increased first and then decreased, and reached the best flexural properties at 64.9%. When the fiber mass fraction is too high, the interlamellar bonding properties will decrease, and the fiber bundles will compress and affect each other. The delamination phenomenon will occur between the layers of the composites, which affects the overall bearing strength and stress limit of the composites. The results of the study show that the composites prepared by VIHPS have excellent mechanical properties, and the content of carbon fiber plays an important role in the influencing factors of the interlaminar and in-layer properties of composites.

## 1. Introduction

Carbon fiber composites have been widely used in aerospace, shipbuilding, construction, mechanical manufacturing and other engineering fields because of their superior specific strength, specific stiffness and designability [1,2,3]. However, the traditional 2D carbon fiber composite lacks fiber reinforcement in the thickness direction, and only relies on the polymer matrix to bear the bonding and load transfer [4]. This leads to poor mechanical properties of the composite in the thickness direction. At the same time, because the strength and modulus of the resin matrix are far lower than that of the fiber, the strength of the interlayer interface is low. The mechanical properties of the composite materials in the direction of thickness are weak, and the composite materials are prone to the failure of interlayer delamination, which will lead to a decrease in the strength and stiffness of the material structure [5,6,7,8]. Therefore, delamination can be a obstacle to the research and development of composites. It is of great significance to study the interlaminar mechanical properties of composites.

In recent years, nanometer materials have played an important role in improving the properties of composites, especially graphene, which has been applied to the field of composite modification by many scholars. Many scholars at home and abroad have devoted considerable research to the improvement of the interlaminar properties and flexural mechanical properties of nano materials. The results have shown that the flexural strength and fracture toughness could be improved by adding GNPs [9,10,11] and GO [12,13] to the epoxy resin matrix, or by chemically grafting GO/ATP onto the surface of T300 carbon fiber [14]. Some scholars have also prepared graphene-reinforced composites by electrophoretic deposition [15], situ polymerization and ethanolic solution precipitation methods [16], and the results show that the flexural strength and fracture toughness of the composites are improved in varying degrees.

During the preparation of composite laminates, the composition ratio of matrix to fiber reinforcer has an important effect on the mechanical properties of composites. Fiber mass fraction is a very important parameter in fiber reinforced composites. Different fiber mass fraction will affect the toughening mechanism and fracture mode of composites. If the content is too low, it will not give full play to the role of reinforcing material, and make it a redundant inclusion or even a source of defects. However, it is easy to form agglomeration and disperse unevenly in the matrix due to the high fiber mass fraction, which cannot bear the effect of force transfer and is not conducive to the improvement of the tensile strength and fracture toughness of the composites. Therefore, reasonable fiber mass fraction is an important part in the design of composites. In the preparation of composites, there can be different types of fiber reinforced materials, which are hybrid composites. The proportion of different components in hybrid composites is an important parameter, which plays an important role in the mechanical properties of composites. Sun [17] conducted tensile and flexure tests on the prefabricated basalt-carbon fiber laminates, proving that the layering sequence has an important effect on the strength and flexure modulus, and the addition of carbon fiber greatly improves the properties of basalt fiber. Yang [18] studied the flexure resistance of six-layer unidirectional composite laminates reinforced by carbon fiber and basalt fiber, proving that the flexure resistance of the composite materials is closely related to the amount of basalt fiber added to the layering. With the increase of basalt fiber content, the flexure modulus of the composite materials decreases. Chandran [19] studied the mechanical properties of composites and found that the mechanical properties of composites increased with the increase of carbon fiber content. The hardness, tensile strength and impact strength of composites with 40% carbon fiber content were superior to other composites. Although many scholars at home and abroad have conducted some studies on the improvement of interlaminar properties and bending mechanical properties of nanomaterials, there are still few studies on the effect of fiber mass fraction on the interlaminar bonding properties of composites, and the estimation of reasonable fiber mass fraction in composites is still not accurate.

In the process of preparing high-performance composites, improper preparation technology may lead to defects in the composites such as cavities, debonding and cracks. It is very important to adopt advanced, scientific and reasonable preparation technology when studying the mechanical properties of composites. At present, the traditional preparation methods of CFRP mainly include resin transfer molding (RTM), winding molding (WM), autoclave molding (AM), and compression molding (CM). The existing preparation process methods have problems of poor infiltration effect, insufficient resin-filled fiber pressure, poor process flexibility and adaptability, and inability to control the preparation process in real time when preparing the composite. In our research, the 2D graphene oxide carbon fiber reinforced epoxy resin matrix (2D CF-GO/EP) composites with different carbon fiber mass fraction were successfully prepared by a vacuum infiltration hot-press forming experimental system (VIHPS) [20] proposed by our team. VIHPS is an advanced new vacuum infiltration hot-press forming process method, which combines the vacuum infiltration process in the RTM and AM process with the molding process in CM process, and realizes the modularization and unified control of the preparation process. The flexural strength and mode II interlaminar fracture toughness (*G*_IIC_) of the composites with different fiber mass fraction were tested by a universal testing machine, and the infiltration microstructure and failure fracture of the composites were analyzed. The effects of fiber mass fraction on the mechanical properties and microstructure of 2D CF-GO/EP were studied. The strengthening mechanism of GO reinforced composites and the influence mechanism of fiber mass fraction on flexural strength and fracture toughness were analyzed, which provided the basis for the performance design of carbon fiber composites.

## 2. Experimental Materials and Methods

### 2.1. Experimental Materials

In this study, the carbon fiber material is 2D-T300 produced by Toray Company, Tokyo, Japan, and its performance parameters are shown in Table 1. The matrix is E-51 epoxy resin (WSR618) produced by Star Synthetic Materials Co., Ltd., Nantong, China. The curing agent is T31 produced by Star Synthetic Materials Co., Ltd., Nantong, China. The graphene oxide (GO) is produced by the improved Hummer method by SuiHeng Technology Co., Ltd., Shenzhen, China, and its performance parameters are shown in Table 2.

### 2.2. Vacuum Infiltration Hot-Press Forming Process

When directly using untreated carbon fibers to prepare the composite, the surface of the carbon fibers is inert and cannot be well bonded with the matrix, and defects such as interfacial cracking and degumming are prone to occur, which ultimately limits the mechanical properties of the composite. Therefore, the preparation process of the epoxy resin mixed solution and the molding process of the composite are very important. Through VIPHS, nano-reinforced materials can be reasonably dispersed in the mixed solution by reasonably controlling the ratio of solvent and process parameters in the mixed solution to improve the binding force between fiber and matrix. In the process of sample preparation, holes, cracks, bubbles and other defects easily form. These problems can be solved by reasonable control of vacuum hot pressing process parameters, and high-performance carbon fiber composites can be obtained.

The whole process of VIHPS can be divided into three parts:

(1) Preparation of the GO dispersion solution

**Figure 1:**
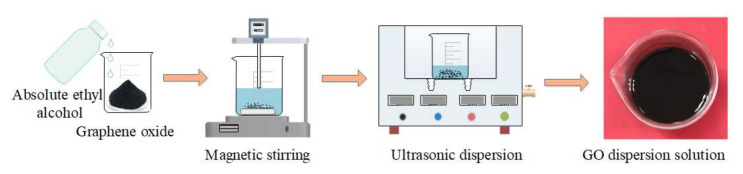
Preparation process of GO dispersion solution.

The solvent of the GO dispersion solution is anhydrous ethanol (AEL). In order to obtain GO with a thin layer and small particle size, electromagnetic stirring and water bath ultrasonic dispersion were used to prepare the GO dispersion. The specific preparation methods are as follows, as shown in Figure 1: (1) GO powder was mixed with an AEL solution, and the mixed solution was stirred by electromagnetic stirring for 30 min at 300 r/min speed. (2) The stirred solution was placed in the water bath ultrasonic dispersion equipment, and ultrasonic dispersion was carried out for 40 min at 40 kHz and 200 W. (3) The lower solution was taken after standing for a little time, and the GO dispersion solution was obtained.

(2) Preparation of the mixed solution of GO and epoxy resin

**Figure 2:**
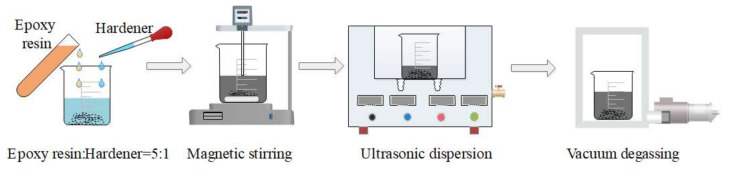
Preparation process of mixed solution of GO and epoxy resin.

The specific preparation methods are as follows, as shown in Figure 2: (1) Quantitative GO and E-44 epoxy resin were weighed by an electronic balance, and the ratio of the GO and E-44epoxy resin was 1:199. (2) E-44 epoxy resin was added to the GO dispersion solution, and the mixed solution was stirred for another 30 min at 300 r/min. (3) The mixed solution was dispersed ultrasonically at 40 kHz and 200 W for 40 min. (4) After standing for a little time, the underlying solution was taken and a 0.5 wt% GO mixed solution was obtained. (5) The mixed solution was placed in a vacuum drying barrel (−0.9 MPa) for 24 h to remove the alcohol and reduce the generation of bubbles, holes and other defects in the prepared composite.

(3) Preparation of GO-CF/EP composite

**Figure 3:**
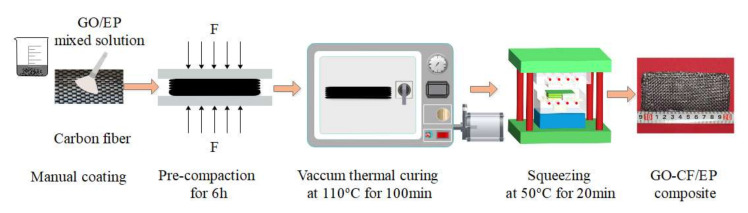
Preparation process of GO-CF/EP composite.

The specific preparation methods are as follows, as shown in Figure 3: (1) a 593 curing agent was added into the mixed solution, in which the proportion of E-44 epoxy resin and 593 curing agent is 4:1, and the mixed solution was stirred by electromagnetic stirring for 10 min at the speed of 500 r/min. (2) The mixed solution was applied evenly on both sides of the carbon fiber cloth, and then the carbon fiber cloths were laminated and preliminarily compacted under a certain pressure. The direction of the adjacent fibers should be strict when stacked. In order to ensure that the pressure will not affect the arrangement of fibers and the dispersion of the resin matrix, the natural compaction of 1000 g iron plate is adopted. (3) After finishing the brushing work, carbon fiber laminates were placed at room temperature for natural curing for 6 h. Sufficient curing time can ensure that the resin matrix can permeate well between the fiber bundles. Then, they were transferred to a constant temperature vacuum drying oven to control the temperature and cure at 110 °C for 100 min. The main purpose of this step was to accelerate the curing of the resin matrix. (4) Carbon fiber laminates were taken out of the constant temperature vacuum drying oven and put into the preheated hot-pressing mold. A certain amount of pressure was applied at 50 °C and hold the pressure for 20 min. (5) Heating and pressurizing was stopped, the spare parts were taken out and the composite was obtained after the mold returned to room temperature.

During the preparation of the GO-CF/EP composite, the size of each group of composite samples should be strictly controlled to ensure that the thickness is 2 mm. In order to strictly control the thickness of the composite samples, we made a mold with a depth of 2 mm, and the samples with different fiber mass fractions were pressed to a thickness of 2 mm using the same pressure during the molding. The number of layers of the carbon fiber cloth in the GO-CF/EP composite was changed. We can obtain the composites with different fiber mass fractions. In order to obtain accurate fiber mass fractions, we accurately measured the mass of CFRP in each group of samples before the test, and then measured the mass of composite samples after the test. The fiber mass and total mass of each group of GO-CF/EP composites were measured. The carbon fiber mass fraction of test samples is 26.6%, 43.3%, 55.5%, 64.9% and 76.4%, respectively.

### 2.3. Testing and Characterization

The instruments and equipment used include scanning electron microscope (SEM), vacuum oven, thermo press machine, bending performance measurement equipment, interlayer fracture toughness measurement equipment, electronic balance, etc. JEOL JSM-6390A SEM was used to observe the impregnation microstructure and flexural fracture morphology of the graphite reinforced composites with different fiber mass fractions.

The DNS100 electronic universal testing machine of the Changchun Mechanical Research Institute was used to test the three-point flexural strength. The test method was in accordance with GB/T 1449–2005. The specimen size is 50 mm × 15 mm × 2 mm, the radius of loading indenter is 5 mm, the span is 40 mm, and the loading rate is 1.0 mm/min. The flexural strength and flexural modulus are calculated by the following formulas:

(1) $$\sigma_{f}=\frac{3P_{max}L}{2bh^{2}}$$

(2) $$E_{f}=\frac{L^{3}\cdot \nabla P}{4bh^{3}\cdot \nabla S}$$

where $\sigma_{f}$ is the flexural strength; *P_max_* is maximum load of the sample; *b* and *h* are the width and thickness of the specimen; l is span of the sample; E is the flexural modulus; ∇P is the load increment; ∇S is the deflection increment.

The mode II interlaminar fracture toughness (*G*_IIC_) of the composites is measured by end notch flexural (ENF) tests. The DNS100 electronic universal testing machine was used, and the test standard is HB 7403-96. The specimen size is 140 mm × 25 mm × 2 mm, the span *2L* is 100 mm, the radius of loading indenter is 5 mm, the radius of support is 5 mm, and the loading rate is 1.0 mm/min. The *G*_IIC_ is calculated by beam analysis theory:

(3) $$G_{\mathrm{IIC}}=\frac{9P_{max}\delta \alpha^{2}}{2W\left(2L^{3}+3\alpha^{3}\right)}$$

where $G_{\mathrm{IIC}}$ is the mode II interlaminar fracture toughness; $P_{max}$ is the crack propagation ultimate load; 2L is the span of the sample; W is the width of the sample; α is the effective crack length; δ is the deflection of the loading point corresponding to the ultimate load.

## 3. Results and Discussion

### 3.1. Dispersion Levels of GO

When the composite is subjected to external forces, the maximum stress is usually located near the interface layer between the carbon fiber and the matrix. After the addition of GO, the structure formed by the internal structure of the composite can increase the maximum stress of the composite to some extent. The presence of GO can also reduce the stress concentration in the material and play a toughening role [21,22]. When the composite is subjected to the pressure load, the dispersion uniformity of GO in the matrix is improved, and the mechanical properties of the composite with better dispersion of graphene are ideal [23].

In theory, the thickness of the GO sheet is only 1 nm, which has a large specific surface area and strong nanometer effect. Therefore, the GO sheet is prone to agglomeration, which significantly reduces the effective specific surface area of the GO sheet, resulting in the failure of the GO sheet to improve the performance of composite materials. In order to obtain the CF-GO/EP composite with excellent performance, it is very important to study the dispersion level of GO sheets in the composites.

**Figure 4:**
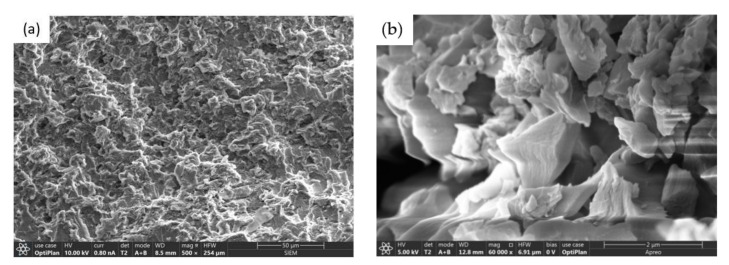
SEM images of GO/EP composites (**a**) GO/EP composites (500×), (**b**) GO sheets (60,000×).

Before analyzing the dispersion levels of GO in the CF–GO/EP composite, the state of GO in the GO/EP composites was observed by SEM at low magnification (500×) and high magnification (60,000×), respectively, to judge the dispersion levels of GO in the epoxy solution. As shown in Figure 4a, with the addition of GO, the GO/EP mixed solution was evenly distributed, and there was no obvious agglomeration of GO in GO/EP. Figure 4b shows the microscopic form of GO. It can be seen that GO is not a flat lamellar structure but a curled structure, which may be due to the introduction of oxygen-containing functional groups after the oxidation of graphite, which weakens the force between the graphite lamellar. This wrinkled surface morphology of the GO nanosheets is expected to increase the friction coefficient of the epoxy resin and can also play an important role in enhancing the interfacial interlocking and the load propagation from the epoxy matrix.

**Figure 5:**
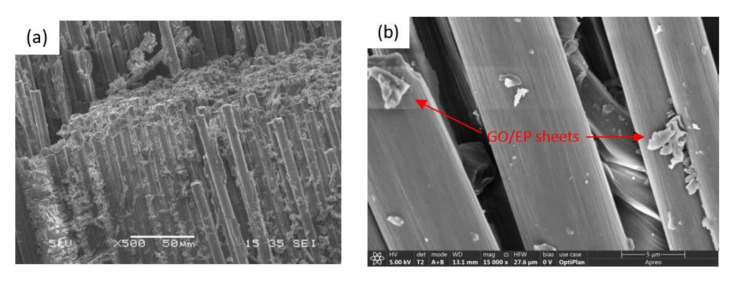
SEM images of CF-GO/EP composites (**a**) 500×, (**b**) 15,000×.

SEM was used to characterize the dispersion level of GO/EP in GO–CF/EP composites at low magnification (500×) first, as shown in Figure 5a. It can be clearly seen from the figure that GO/EP is distributed randomly among the fiber bundles, indicating that GO/EP particles infiltrate evenly in the composite and the dispersion effect is relatively ideal. After GO in the resin matrix, there is no side effect on the properties of the composite due to the aggregation of graphene particles, thus affecting the accuracy of the test results. In order to further characterize the dispersion degree of GO/EP in the GO–CF/EP composite, the microscopic image of GO/EP in the composite was obtained by SEM under high magnification, as shown in Figure 5b. it is apparent that GO/EP sheets are attached to the surfaces of CF bundles, as indicated by the red arrows in Figure 5b. Therefore, it can be proven that the substances in Figure 5a are GO/EP sheets rather than agglomerated GO/EP particles.

### 3.2. Flexural Property

When the composite is subjected to flexural load, the local stress concentration occurs due to the interface and micro-crack defects in the middle layer of the material. When the microcrack tip of some fibers has concentrated energy, the crack will be further expanded. If this energy is large enough, the stress intensity factor reaches a critical value, and the cracks lose stability and will extend and expand, which then leads to interlocking fracture of the fibers and brittle failure of the composite. For 2D braided composites, on the one hand, braided fiber bundles can change the direction of crack propagation and increase the stress intensity factor of the composites. On the other hand, fiber bundles with different directions help to prevent the vertical propagation of fracture cracks. In order to study the influence of fiber mass fraction on the flexural properties of composites, the three-point flexural test was carried out. 

**Figure 6:**
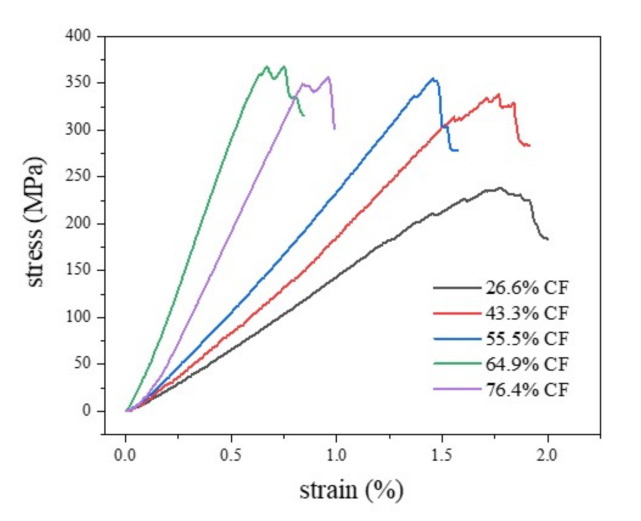
Stress–strain diagram of three-point flexural test.

Figure 6 shows the flexural stress–strain curves of test materials with different carbon fiber mass fractions in the three-point flexural test. It can be seen from Figure 6 that the curve changes from 64.9% CF to 26.6% CF, from steep to gentle gradually, and the maximum stress value decreases gradually. Compared with other curves, 64.9% CF has the highest stress value, and 26.6% CF has the lowest stress value. This indicates that in the five groups of three-point flexural tests, the sample with 64.9% content has the highest flexural strength, while the composite with 26.6% content has the lowest flexural strength.

**Figure 7:**
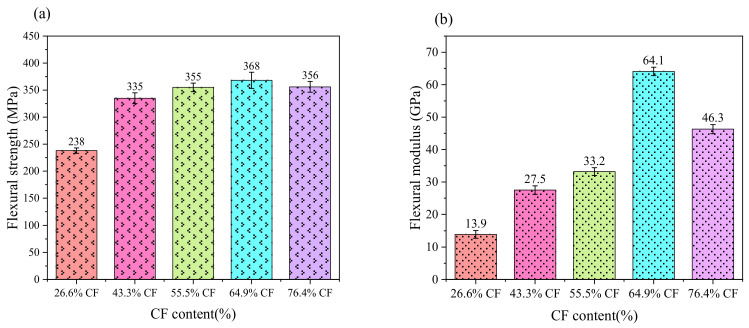
The test results of three-point bending test (**a**) flexural strength, (**b**) flexural modulus.

The results of each group test were repeated three times and the average value was obtained. Figure 7 shows the average value of flexural strength and the flexural modulus values of specimens with different fiber mass fractions. It can be seen from Figure 7 that the flexural strength and flexural modulus of the composite with 64.9% content are the highest. When the content of carbon fiber increases from 26.6% to 76.4%, the flexural strength and flexural modulus of the composite show the same change trend. When the fiber mass fraction increases from 26.6% to 64.9%, the flexural strength and flexural modulus increase gradually with the increase of the fiber mass fraction, and the maximum increases are 54.6% and 361.2%, respectively. When the fiber mass fraction exceeds 64.9%, the flexural strength and flexural modulus show a decreasing trend. As can be seen from Figure 7, the strength of the composite was improved because the GO could effectively connect the interface layer between the carbon fiber and epoxy resin matrix after adding 0.5wt% GO. Therefore, the flexural strength of the five groups of composite laminates with different fiber mass fractions was improved to varying degrees compared with the original composite.

Elastic modulus is an index reflecting the ability of a material to resist elastic deformation. The greater the modulus, the greater the rigidity of the material, and the greater the stress required by a certain elastic deformation of the material. With the increase of carbon fiber mass fraction, the flexural modulus and the flexural strength will increase greatly. However, the flexural strength and the flexural modulus will show a downward trend when the fiber mass fraction exceeds 64.9%. This shows that an increase of fiber mass fraction will lead to the extrusion and influence of carbon fiber when the fiber mass fraction is higher than 64.9%. The resin matrix between the fiber bundles is too low, which will affect the overall bearing strength and stress limit of the composite. The flexural strength and flexural modulus of the composites will increase with the increase of carbon fiber mass fraction. However, when the content of carbon fiber reaches 64.9% and the content of carbon fiber in the composite laminates continues to increase, the interlaminar properties of the composite laminates will decline sharply because of the decrease of resin matrix, and the transverse bearing capacity of composites will decrease significantly. Therefore, the flexural strength and flexural modulus will decrease.

### 3.3. Interlayer Fracture Toughness

The measured *G*_II__C_ is considered to represent the critical strain energy release rate during crack preforming and crack propagation. In this study, the crack propagation is caused by the load generated by flexural stress, and the crack propagation occurs under the action of shear force at the crack tip. The interfacial strength of the fiber and matrix has an important effect on the mechanical properties of the composites. When fiber reinforced composites fracture, cracks mainly spread along the interface between the fiber and matrix. When the interfacial strength is good, the fiber and matrix are not easy to debonding, and more matrixes will adhere to the fiber surface [24].

**Figure 8:**
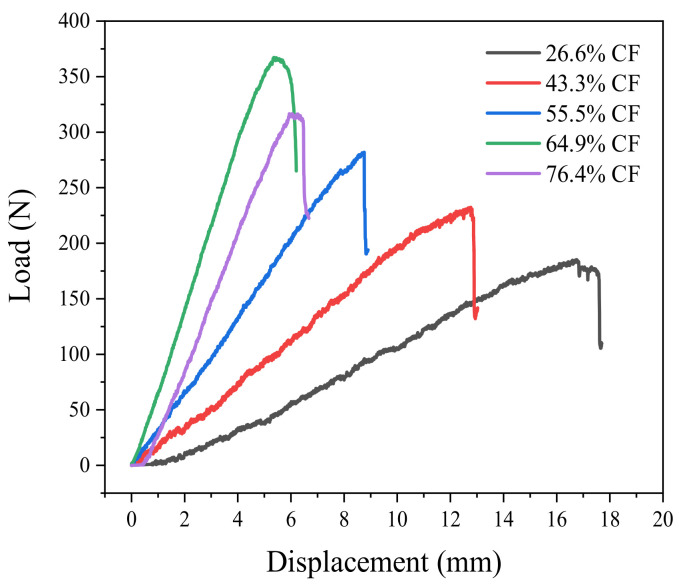
Load-deflection curve of the ENF test.

Figure 8 shows the load-displacement curve of the ENF test, and the curve given is the test sample closest to the average value of repeated samples. It is clear from Figure 8 that the maximum load of 64.9% CF specimen is higher than that of other specimens, while the deflection is lower than that of other specimens. The load-deflection curves of 55.5% CF, 64.9% CF and 76.4% CF are linear, reflecting the brittleness of the matrix resin. When the maximum load is reached, the load drops sharply and cracks become unstable and spread.

**Figure 9:**
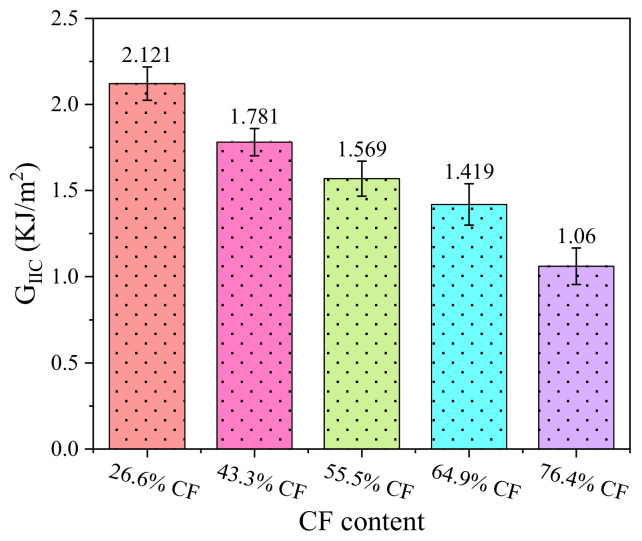
*G*_IIC_ of composite with different fiber mass fraction.

**Figure 10:**
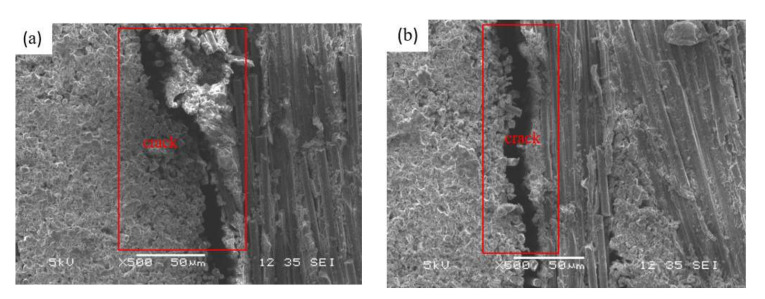
SEM image of crack propagation (500×). (**a**) 26.6% CF, (**b**) 76.4% CF.

The results of each group test were repeated three times and the average value was obtained. Figure 9 is the calculated average value of *G*_IIC_. As can be seen from Figure 9, when the carbon fiber mass fraction increases from 26.6% to 76.4%, the composite of the *G*_IIC_ presents an obvious decreasing trend. When the fiber mass fraction reached 64.9%, the *G*_IIC_ sharply declined with the increase of the fiber mass fraction. The interlaminar crack propagation directions with 26.6% and 76.4% fiber mass fractions in Figure 10 were observed and analyzed. When the sample with a 26.6% fiber mass fraction is subjected to bending load, the prefabricated cracks will extend to the surrounding composite layers in a wide range. However, when the sample with a 76.4% fiber mass fraction is subjected to a bending load, the crack propagation to the surrounding composite layer is very limited. When the fiber content in the composite increases gradually, the content of the epoxy resin matrix and GO decreases gradually, and the enhancement effect of the GO on the interlaminar bonding property of the composite is weakened, so the interlaminar fracture toughness of the composite with high fiber content is weak. When the content of the carbon fiber increases gradually, the bond between the composite layer and the adjacent composite layer fiber bundle is weak due to the high content of carbon fiber. Under the bending load, the prefabricated cracks between the fiber layers will expand rapidly between the composite layers, and the flexural bearing capacity of the composite will decrease.

### 3.4. Fracture Morphology Analysis

In carbon fiber reinforced resin composites, carbon fiber with high strength and high modulus plays a major bearing role, providing structural stiffness and strength and controlling its basic properties, while matrix resin materials support and fix the fibers [25]. The interface between the fiber and resin plays a very key role in this process. The interface transfers the load applied on the matrix material to the fiber as the reinforcing material and disperses and transfers the load between the fibers. Therefore, changing the interface condition will also affect the damage mode [26].

**Figure 11:**
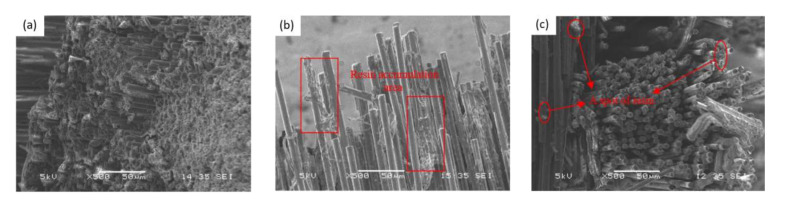
Three-point flexural microfracture of composite (500×). (**a**) 26.6% CF, (**b**) 64.9% CF, (**c**) 76.4% CF.

In order to investigate the influence of fiber mass fraction on the flexural strength and the mechanism of action, the flexural fracture morphology of composites with different fiber mass fractions was analyzed. Figure 11 shows the microstructure morphology of the flexural fracture of composites with 26.6%, 64.9% and 76.4% fiber mass fraction, respectively. As can be seen from Figure 11, there are neat fracture and fiber pull-out phenomena. Some fibers are broken, and the other part is pulled out, and no obvious defects are found. The fracture morphology of the composite is ideal. A large amount of resin was filled in the gap between the fiber bundles, which was relatively full and uniform. No obvious holes or other defects were found. This indicates that the resin penetration effect of the five groups of materials was relatively ideal. As can be seen from Figure 11a, there are a large number of resins in each area of the impregnation microstructure, and the distribution is relatively uniform. The fiber bundles were compactly stacked, and no obvious void defects were found. There is a lot of resin retained on the fiber layer, and the combination of resin and fiber is good, which indicates that the impregnation effect of the composite is good. The infiltration effect of composites in Figure 11b,c are slightly worse than those of the composites in Figure 11a. In the impregnation zone, there is still sufficient resin distribution on the carbon fiber. However, in the impregnation zone, the impregnation effect is poor, and there is only a small amount of resin impregnation into the gap of the carbon fiber. At the same time, the curved fracture surface of the composite is uneven, and there are many holes left by the carbon fiber pulling out and protruding broken fiber bundles. This indicates that the carbon fiber begins to break due to the load during the fracture process of the sample under the flexural load. As the bond between the carbon fiber and the resin is very close, part of the carbon fiber is pulled and leads to the separation of the fiber and the resin. The increase of fiber mass fraction leads to a decrease of the epoxy resin matrix in the composite, so the resin content and resin distribution in the microscopic figure have obvious changes. The 26.6% composite with the lowest fiber mass fraction has a large number of resin matrixes with uniform distribution peaks. However, the distribution of fiber bundles was scattered in the composite with the highest fiber mass fraction of 76.4%. Only a small amount of resin distribution could be seen, and the resin matrix agglomeration phenomenon was also present. The impregnation effect of the composite with 76.4% content is not ideal.

Epoxy resin is an important type of thermosetting polymer matrix, and GO is a commonly used filler for toughening fiber reinforced composites [27]. Many researchers have studied the toughening mechanism of GO [28,29,30]. However, in the case of the same interlaminar toughening material, it is still necessary to make great efforts to further improve the mode II interlaminar fracture toughness and the underlying mechanism of the fiber reinforced epoxy composite. Figure 5 shows the good dispersion of the mixed solution of GO in epoxy resin in the carbon fiber composites. GO in the epoxy matrix has good dispersion, and the interface adhesion and stress distribution uniformity of the composite are good. The stress concentration phenomenon is improved, and the mechanical properties of the composites are improved.

**Figure 12:**
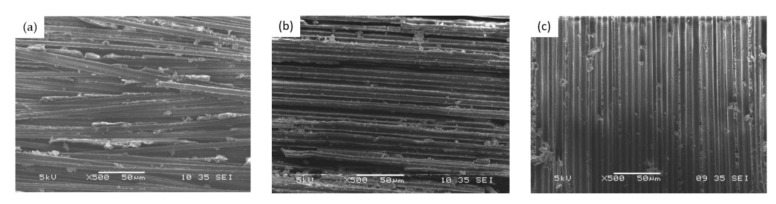
ENF test microfracture of composite (500×). (**a**) 26.6% CF, (**b**) 64.9% CF, (**c**) 76.4% CF.

Figure 12 shows the ENF test microfracture of the composite with different fiber mass fractions. In the microscopic diagrams of the interlaminar interface of the three groups of samples, from Figure 12a–c, it can be clearly seen that the distribution of the epoxy resin matrix at the interlaminar interface is increasingly sparse. In Figure 12a, a large number of epoxy resin matrixes are wrapped between the fiber bundles, which are relatively loose and evenly dispersed among the fiber bundles. It can be seen from Figure 12b that with the increase of the carbon fiber mass fraction, the fiber bundles are compact and neat, with only a small amount of matrix dispersion between the fiber bundles. As can be seen from Figure 12c that when the fiber content is increased to 76.4%, the matrix content in the composite is very small, and the matrix will appear uneven in dispersion.

Compared with other samples, the fracture surface of samples with low carbon fiber mass fraction is rougher and more broken. This shows that the flexural load caused rich resin matrix cracking on the surface of the local area. A large number of resins and GO have improved the interfacial interaction between the matrix and fiber, and the enhancement effect from nanometer to macroscopic is produced in the resin-rich zone between laminates. At the fracture interface, some fibers can be seen to cluster together, and the fibers are interlaced with each other. The contact between the fibers enhances the resistance to the tearing of the layers. However, the compact fiber bundle accumulation weakens the matrix’s ability to infiltrate the gap with the increase of the carbon fiber mass fraction. When the fiber mass fraction is lower, it can be seen that the gap between the fiber bundles at the fracture interface is larger. When the content of epoxy resin was less, the carbon fiber in the sample unstuck with the matrix. This indicates that the interfacial bond between the fiber and the matrix was poor. Therefore, with the increase of the carbon fiber mass fraction, the interlaminar fracture toughness of the composite laminates becomes smaller and smaller, and the *G*_IIC_ decreases with the increase of the fiber mass fraction.

## 4. Conclusions

In this paper, five groups of 2D CF-GO/EP with 26.6%, 43.3%, 55.5%, 64.9% and 76.4% fiber mass fraction were prepared by VIHPS. Three-point flexural tests and ENF tests were carried out.

(1) Based on a new vacuum pressure infiltration CFRP method, 2D graphene oxide carbon fiber reinforced epoxy resin matrix (2D CF-GO/EP) composites were prepared, and the microstructure of the CF–GO/EP composites and GO/EP composites was observed by scanning electron microscopy. The results showed that the GO was evenly dispersed in the composites, and the impregnation effect of the resin matrix was also ideal. The flexural and fracture resistance of the composites were improved after the addition of GO.
(2) The three-point flexural test results show that the flexural strength and flexural modulus of the composites with different fiber mass fractions show the same changing trend. With the increase of fiber mass fraction from 26.6% to 76.4%, the flexural strength and flexural modulus increase first and then decrease and reach the maximum value at 64.9%. The main reason is that too high fiber mass fraction will lead to the mutual extrusion and influence of carbon fibers, and too low epoxy matrix between fiber bundles will reduce the interlaminar bonding performance of the composites. Therefore, there will be delamination between composite layers, which will affect the overall bearing strength and stress limit of the composites.
(3) ENF test results show that with the increase of fiber mass fraction in the composite laminated plate, mode Ⅱ interlaminar fracture toughness (*G*_IIC_) gradually decreases, and 26.6% content of carbon fiber composite has strong interlayer bonding properties. When the carbon fiber mass fraction increased to 76.4%, the *G*_IIC_ was reduced by 50%. When the fiber mass fraction increases gradually, the bond between fiber bundles becomes weak. When the composite is subjected to a bending load, the prefabricated cracks propagate rapidly, and the composite shows small fracture toughness.

## Figures and Tables

**Table 1:** Physical properties of T300 carbon fibers.

Fiber Type	Fiber Diameter of the Monofilament (10^−6^ m)	Ultimate Tensile Strength (MPa)	Young’s Modulus (GPa)	Elongation (%)	Density (g/cm^3^)
T300	6~8	3500	230	1.5	1.76

**Table 2:** Material parameters of GO.

Purity	Layers	Thickness	Diameter	Carbon Content	Oxygen Content	Sulfur Content	Stripping Rate
99%	1~2	1 nm	0.2~10 um	<46%	>48%	<1.5%	96%

## Data Availability

Data are available within the article.
